# High-load resistance training and FSHD: harmful mixture or a silver bullet combination?

**DOI:** 10.1007/s00415-026-14148-7

**Published:** 2026-09-18

**Authors:** Marius Meinhold, Troy A. Hornberger

**Affiliations:** 1 Department of Comparative Biosciences, University of Wisconsin-Madison, Madison, WI USA; 2 School of Veterinary Medicine, University of Wisconsin-Madison, Madison, WI USA

**Keywords:** Facioscapulohumeral muscular dystrophy, Exercise, Neuromuscular disease, Overwork weakness, Muscle damage, Muscle hypertrophy

## Abstract

Facioscapulohumeral muscular dystrophy (FSHD) is a progressive neuromuscular disorder characterized by asymmetric loss of muscle mass and strength, as well as fatty infiltration, inflammation, oxidative stress, and fibrosis. Unfortunately, as the disease progresses, these features can lead to the loss of mobility and independence. High-load resistance training (RT) is a well-established therapeutic strategy for increasing muscle mass and strength, and emerging evidence suggests it may also attenuate fatty infiltration, inflammation, oxidative stress, and fibrosis in other populations. Despite this therapeutic potential, high-load RT has long been approached with caution in FSHD, and current recommendations largely discourage its use. In this review, we critically examine the historical and scientific basis for this dogma and find that it rests on a small number of poorly substantiated and largely unreplicated studies. We further show that the limited RT trials conducted in individuals with FSHD have consistently reported good tolerability and, in several cases, moderate improvements in strength and function. Critically, no study to date has implemented a true high-load RT protocol alongside validated markers of muscle damage, a design that accounts for the asymmetry of the disease, and imaging that is sensitive enough to detect disease-modifying effects (e.g., MRI). We argue that the current consensus against high-load RT in FSHD reflects an absence of evidence rather than evidence of no benefit. Thus, we hope this narrative review encourages the FSHD community to revisit fundamental questions about the safety and therapeutic potential of high-load RT, and to create practical guidelines based on rigorous studies.

## Introduction

Facioscapulohumeral muscular dystrophy (FSHD) is one of the most frequent forms of hereditary and progressive neuromuscular disorders (NMDs) [1]. As the name implies, the disease primarily affects the muscles of the face, scapula, and upper arms. A genetic model proposes that individuals with FSHD have a contraction of the D4Z4 macrosatellite repeats on chromosome 4q35, which causes hypomethylation of the region and leads to sporadic expression of the myotoxic double homeobox *DUX4* gene [2, 3]. In combination with specific single-nucleotide polymorphisms in the chromosome region distal to the last DZ4Z repeat, this induces polyadenylation of *DUX4* transcripts, which allows for subsequent synthesis of the protein [4]. Individuals with this specific genetic background are diagnosed with FSHD1 and represent the vast majority of cases [5]. In contrast, about 5% of all cases lack a contraction on chromosome 4q35, but still show a loss of methylation and heterochromatin markers of D4Z4 repeats, resulting in sporadic expression of DUX4. Individuals with this genetic background are diagnosed with FSHD2 [6]. As a transcription factor, DUX4 regulates the expression of several target genes associated with germline and early stem cell development that are normally silent in mature skeletal muscle [7]. While the distinct mechanisms are not well understood, it is believed that the aberrant expression of these target genes contributes to the loss of cellular homeostasis, as well as the induction of inflammation and apoptosis [7]. Despite differences in genetic background, the clinical presentation appears largely identical between the two forms of the disease [8].

Recent estimates suggest that the current prevalence of FSHD is 5 per 100,000 individuals, with no clear differences between males and females [1, 9, 10]. In contrast to other NMDs, such as Duchenne muscular dystrophy (DMD), individuals living with FSHD have a normal life expectancy [11, 12]. Given global demographic changes and the ongoing increase in life expectancy, it is reasonable to assume that the prevalence of FSHD will rise in the future [13, 14]. This will present a substantial socioeconomic burden, exacerbated by the progressive nature of FSHD. For instance, a recent study found that individuals with FSHD in the Netherlands incur health care-related costs that are 5 times higher than the mean per capita costs of healthy individuals, and one of the major driving factors of these costs is the progressive decline in mobility [15, 16].

In line with the decline in mobility, individuals with moderate-to-severe FSHD also experience marked reductions in quality of life [17, 18]. Fortunately, several DNA- and RNA-based therapeutics are currently in development. However, previous approaches to treat FSHD, such as anti-inflammatory or antioxidant compounds, have demonstrated limited or no success [12, 19]. Hence, there is an urgent need to develop cost-effective therapies capable of altering disease progression [20]. In a recent review, Crisafulli et al. appraised the evidence for physical exercise as a non-pharmacological therapy and highlighted the potential benefits of endurance-oriented training for individuals with FSHD [21]. High-load resistance training (RT) represents another promising option given its well-established capacity to induce robust increases in skeletal muscle mass and strength [22]. However, despite its therapeutic potential, the use of high-load RT in individuals with FSHD has long been approached with caution—even skepticism—driven in part by persistent misconceptions about its effects. Accordingly, the purpose of this narrative review is to: (1) summarize the pathological features of FSHD; (2) familiarize readers with the principles of high-load RT; (3) critically appraise the prevailing dogma surrounding high-load RT in individuals with FSHD; and (4) review the evidence that high-load RT ameliorates the major pathological features of the disease in other populations.

## The pathological features of FSHD

The major pathological features of FSHD include a progressive and asymmetric loss of skeletal muscle mass, along with fatty infiltration, inflammation, oxidative stress, and fibrosis within the affected muscles [23]. Together, these features ultimately lead to a progressive decline in strength and mobility [23]. Many of the symptoms can go clinically unrecognized for several years, and this likely contributes to the highly variable age of onset that is characteristic of the disease [24, 25]. Additionally, the severity and progression of FSHD vary extensively, with up to 30% of carriers reporting no symptoms and approximately 20% becoming wheelchair-dependent [26, 27].

At the whole-body level, the loss of skeletal mass is relatively modest, as suggested by a 16% lower whole-body lean mass, which correlates well with skeletal muscle mass, compared to age-matched controls [28–31]. However, consistent with the differential involvement of muscles that is characteristic of the disease, regional lean mass in the arms and thighs can be 25% lower than in healthy, age-matched controls [28–31]. Of note, these values were obtained using dual-energy X-ray absorptiometry, which is insensitive to fatty infiltration and might, therefore, overestimate skeletal muscle mass in individuals with FSHD who have pronounced fatty infiltration [32, 33].

As alluded to above, the loss of muscle mass is frequently accompanied by severe, asymmetric fatty infiltration, which is primarily detected using magnetic resonance imaging (MRI) [34]. For instance, Dahlqvist et al. reported MRI-based fat fractions that were more than 2–3 times greater in individuals with FSHD than in age-matched controls [35]. In addition, fatty infiltration is not uniform throughout the muscle, but usually increases from proximal to distal within the affected muscles [36–39]. Given that several studies reported significant correlations between fatty infiltration and measures of strength and physical function, this radiological feature is often viewed as an informative marker of disease progression [34, 36, 40–42].

Quantitative MRI has further revealed the presence of short tau inversion recovery (STIR) hyperintensity lesions in the muscles of individuals with FSHD [34, 37, 43, 44]. These lesions reflect increased water content (i.e., edema) and are typically interpreted as signs of inflammation [45]. Consistent with this interpretation, the presence of STIR-positive lesions is associated with elevated levels of inflammatory markers in muscle biopsies, such as mononuclear cell infiltrates and the expression of the major histocompatibility complex I [43, 46–49]. Additionally, individuals with FSHD exhibit higher circulating levels of activated immune cells and several pro-inflammatory cytokines, including TNFα, RANTES, IFNα2, MCP-1, and IL-6 [43, 50, 51]. Collectively, these findings indicate that a pronounced inflammatory response is a common pathological feature of FSHD.

In line with the inflammatory response, several studies indicate that oxidative stress is another pathological feature of the disease [51–54]. For instance, transcriptomic analyses of biopsies from individuals with FSHD revealed significant upregulation of genes annotated to the activation of the oxidative stress-associated TNFα pathway [52]. This is further supported by an increased susceptibility to oxidative stress found in human FSHD myoblasts [53]. In addition, several markers of systemic and local oxidative stress were elevated in individuals with FSHD [51]. This includes a decrease in the reduced to oxidized glutathione ratio (i.e., an accumulation of oxidized glutathione) in the blood, as well as a greater number of lipofuscin inclusions in biopsies from the vastus lateralis [51].

Histopathological analyses further revealed that muscles with STIR-positive lesions also exhibit greater collagen deposition (i.e., fibrosis) than muscles that lack these lesions [44]. Likewise, muscles with STIR-positive lesions also have a higher number of non-myogenic interstitial cells and higher expression of fibrosis-associated genes [55]. Unsurprisingly, fibrotic muscles also show signs of regeneration, such as an increase in the number of muscle fibers with central nuclei, and the presence of muscle fibers that stain positive for embryonic myosin heavy chain [56]. In addition to markers of muscle fiber regeneration, muscles affected by FSHD were shown to have decreased capillary density and elevated counts of apoptotic nuclei, which supports the notion of impaired tissue homeostasis [57].

Importantly, the pathological features described above likely accumulate progressively, as demonstrated by several longer-term follow-up studies for some of these features. Radiological features represent the most sensitive markers of disease progression, including increases in fatty infiltration and the abundance of STIR-positive lesions, and a decrease in muscle volume within 1 year [37, 41, 58–61]. The increases in fatty infiltration are mostly detectable in muscles that show STIR-positive lesions or some fatty infiltration at baseline, which suggests that already affected muscles might be most vulnerable to further deterioration [37, 41, 58]. Functional outcomes and clinical scores, however, can remain largely unchanged over this period or progress at rates that do not correlate with those of the radiological features [41, 59–62]. This raises the question of whether more sensitive measurements are necessary to appropriately monitor the functional decline or increases in clinical severity.

As mentioned previously, the accumulation of the pathological features is associated with asymmetric muscle weakness predominantly in the face, the shoulder girdle, and the upper arms [28, 63, 64]. The muscles of the trunk and lower limbs are also severely affected in most cases, leading to loss of ambulation in the later stages of the disease [23, 35, 40, 41, 65]. Compared with age-matched controls, individuals with FSHD consistently show reductions in maximal voluntary contraction (MVC) force of >40% [51, 66–68]. This loss of strength exceeds that predicted from the loss of muscle mass alone, indicating that the force per cross-sectional area (CSA; i.e., specific force) is also reduced [66, 67]. Interestingly, even when correcting the total CSA for fatty infiltration (i.e., normalizing to contractile CSA), Lassche et al. found a reduction in specific force of >30% [66]. However, a previous study by the same group also showed that the maximum specific force of isolated single muscle fibers from the same individuals with FSHD is very similar to that observed in healthy, age-matched controls [69]. This is a noteworthy point because it suggests the decline in the specific force during MVC’s is due to impairments in the collective force-generating capacity of the muscle and/or impaired neural activation.

When considering the above possibilities, it is important to recognize that, at the whole-muscle level, the collective force-generating capacity is strongly influenced by the CSA of the individual muscle fibers and the total number of muscle fibers. Yet, the changes that occur at the level of the muscle fibers in FSHD remain poorly defined. For instance, only a few studies have quantified fiber size and, surprisingly, most of the studies pointed towards an increase in the mean fiber size in individuals with FSHD [49, 69, 70]. However, these studies were limited by the small number of fibers analyzed [69], the small number of subjects [70], unclear blinding conditions during the analyses [49, 69, 70], or the quantification of fiber diameter [49, 70]. The latter point is worth highlighting because previous studies have reported the presence of small angular fibers in muscles affected by FSHD, and thus, changes in fiber diameter may not be reflective of equivalent changes in fiber CSA [71, 72]. Therefore, in our opinion, the available evidence regarding the effects of FSHD on fiber CSA remains inconclusive.

This uncertainty is important to keep in mind because reductions in whole-muscle force could arise not only from changes in fiber size, but also from changes in the total number of muscle fibers. Indeed, it has previously been shown that the contractile CSA is markedly reduced in severely affected individuals [66]. Since prior findings about fiber CSA remain inconclusive, a key question emerges: Does FSHD lead to muscle fiber loss? To date, several lines of indirect evidence have suggested that this may indeed be the case. For instance, FSHD leads to increased markers of muscle fiber regeneration, decreased capillary density, accumulation of non-myogenic interstitial cells, and elevated counts of apoptotic nuclei, which are reminiscent of other muscular dystrophies in which there is strong evidence for muscle fiber loss [48, 55–57, 73]. Additionally, muscle fiber loss in other muscular dystrophies, such as DMD, is associated with the exhaustion of the regenerative capacity of skeletal muscle [74, 75]. Likewise, several lines of evidence suggest that primary myoblasts extracted from muscle biopsies from individuals with FSHD may exhibit impaired myogenic differentiation, which potentially reduces the regenerative capacity of muscles affected by FSHD [53, 76–79]. Together with the accumulation of fibrotic tissue, which is thought to replace lost muscle fibers, this could indicate a mismatch between the degenerative and regenerative processes in FSHD [44]. However, we are not aware of any studies that have directly addressed whether there is a net loss of muscle fibers in FSHD.

On this note, it is also worth considering that the pathological features of FSHD, such as fibrosis, edema, and the accumulation of non-myogenic interstitial cells, would further decrease the density of contractile tissue. At first glance, this would appear to contrast with the work of Lassche et al., which reported a decrease in specific force even when correcting for fatty infiltration [66]. However, MRI-based assessments of muscle composition generally do not capture changes in fibrosis, edema, and interstitial cells [44, 66, 80]. As such, these features were likely included in the "contractile" CSA estimates used by Lassche et al. Put differently, the "contractile" CSA measurements reported by Lassche et al. may have overestimated the area that was actually occupied by muscle fibers and, in turn, contributed to the apparent reduction in specific force that they observed [66].

In addition to the aforementioned factors, impairments in neural activation could also contribute to the reduced specific force. Specifically, there is currently no definitive evidence to support impairments at the supraspinal or spinal level; however, several lines of evidence suggest that there could be defects in excitation–contraction (EC) coupling [66, 67, 81–89]. For instance, a recent meta-analysis revealed downregulation of genes associated with neuromuscular junction architecture, raising the possibility that the transmission of action potentials to muscle fibers could be compromised [86]. In line with this possibility, Stübgen applied single-fiber electromyography to the triceps brachii muscles of individuals with FSHD and found a significant correlation between electromyographic jitter, a marker of abnormal neuromuscular transmission, and disease duration, which suggested progressive alterations of the neuromuscular junction [81]. Sarcolemmal reorganization has also been reported in muscles affected by FSHD, as evidenced by an increased distance between the sarcolemma and the nearest myofibril [87, 88]. This is notable because it could disrupt the T-tubule network and impair the transmission of depolarization signals to the sarcoplasmic reticulum. In addition, muscle excitability studies performed by Lycett et al. provided evidence of relative depolarization of the resting membrane potential in muscle fibers from individuals with FSHD [89]. Such depolarization could lead to partial inactivation of voltage-gated sodium channels, thereby reducing action potential propagation and attenuating Ca^2+^ release from the sarcoplasmic reticulum [90]. Thus, when taken together, there appears to be consistent support for the notion that impaired EC coupling contributes to the reduction of specific force that is observed in FSHD.

In summary, FSHD is characterized by progressive, asymmetric muscle weakness that appears to arise from a combination of well-established pathological features, including muscle loss, fatty infiltration, inflammation, oxidative stress, fibrosis, and, as hypothesized above, potentially also from impairments in excitation–contraction coupling. Recognizing these features is essential for the remainder of this review because each represents a potential target of high-load RT and, in turn, shapes the criteria by which its therapeutic value should be judged. Specifically, if high-load RT is to meaningfully alter the course of FSHD, it should not only counter the loss of muscle mass and strength, but also favorably affect fatty infiltration, inflammation, oxidative stress, and fibrosis, thereby potentially delaying disease progression or reducing disease severity. Furthermore, the asymmetry and inter-individual variability that characterize the disease must be carefully considered when designing, conducting, and interpreting RT studies in this population.

## High-load resistance training: definition and primary outcomes

### What is high-load resistance training?

Before addressing the current dogma surrounding high-load RT and its potential benefits for individuals with FSHD, it is important to define what constitutes high-load RT and to review the acute and long-term responses that it elicits. In general, RT programs are characterized by several key variables that can be manipulated to optimize training outcomes (Table 1) [91]. This includes the load, volume, rest intervals, frequency, and progressive overload [22]. In the following chapter, we will first cover the key variables that define a single bout of RT, followed by the variables that concern the long-term nature of RT.

**Table 1:** Summary of the variables that define a high-load RT program and the respective recommendations that have been established in healthy populations

Variable	Definition	Recommended for hypertrophy & strength
Load	The weight that is lifted; expressed as a percentage of the highest load one can lift once (i.e., one-repetition maximum (1RM)) or as repetition maxima (e.g., 8RM)	70–90% 1RM or 4–12RM
Volume	Total number of sets in the targeted load range with a single set defined as the intent to execute the target number of repetitions uninterrupted (e.g., 8 repetitions for an 8RM)	4–10+ sets per muscle group per week
Rest interval	Recovery time between sets and exercises	1–2+ minutes
Frequency	Number of bouts (i.e., training sessions) per week	2–3+
Progressive Overload	Gradual increase of the stimulus in terms of load, volume, and/or frequency	Increase load to maintain targeted repetition range

The first variable, the load, mainly distinguishes RT from endurance training, in which the load is much lower. Historically, loads are commonly prescribed as a percentage of an individual’s 1RM, a nomenclature adopted from elite weightlifting [92]. While it seems intuitive and is still frequently used in scientific literature, this approach has two major limitations when applied to other populations. First, it relies on the valid and reliable assessment of the 1RM for each exercise, which may not be feasible for some individuals [93–96]. Second, several studies have demonstrated that the number of repetitions performed at a given percentage of their 1RM varies considerably between individuals and between exercises within an individual [97–100]. Alternatively, as applied in many studies and in practice, loads can be prescribed as repetition maxima. For instance, a 10RM is the highest load an individual can lift exactly 10 times, which roughly equates to 80% 1RM [97–101]. Thus, for comprehensiveness, we will provide a definition for high-load RT that encompasses both concepts, but we encourage the application of repetition maxima in research, as well as in practice.

In its simplest form, RT programs are defined by repeated muscle contractions against progressively greater loads [22]. This definition begs the question of what constitutes a high-load RT program. While there is no consensus on what constitutes “high”, loads above 70–80% 1RM or 10–12RM are commonly referred to as high loads. In the past, many authors argued that loads of ≥60% 1RM or ≥15-20RM are necessary to elicit robust increases in muscle mass (i.e., hypertrophy) in healthy adults [102–106]. However, more recent meta-analyses have concluded that loads of <60% 1RM or <15-20RM result in comparable muscle hypertrophy when training volume is equated across groups [107–110]. That said, previous studies have also consistently shown that the use of higher loads leads to greater increases in maximal strength [107–112]. Therefore, in this review, we will define high-load RT as any RT program that utilizes loads in the range of 70–90% 1RM or 4–12RM, as these loads appear to be the most effective at simultaneously increasing muscle mass and strength [22].

Having defined the load range that characterizes high-load RT, the next variable to consider is training volume. This is an important variable because the literature suggests that performing multiple fatiguing sets per muscle group per week is superior to a single set per week to enhance training responses [91, 102, 110]. Indeed, in its latest position stance, the American College of Sports Medicine concluded that training volume is the main variable that enhances muscle hypertrophy responses [22]. While the minimum effective volume for detectable hypertrophy is estimated to be around 4 sets per muscle group per week, a weekly volume of 10 sets or more per muscle group appears to be beneficial for maximizing hypertrophy in healthy individuals [22, 113]. This is considerably more than the 2–3 sets/exercise that seems sufficient to maximize strength responses [22, 113]. Thus, practitioners should aim for a volume of at least 4 near-maximal-effort sets per muscle group per week to simultaneously induce hypertrophy and increases in strength [22, 113].

When training with the minimum volume, it may be particularly important to perform all sets with maximal effort and movement quality to elicit robust training responses. To ensure that this is feasible, individual sets should be separated by sufficient rest. While there is no clear advantage for training responses with rest intervals of ≥1 min, it should be noted that shorter rest intervals are associated with higher perceived exertion [91, 114–118]. Higher perceived exertion can pose a challenge to maintaining effort and movement quality, particularly among less experienced individuals. Thus, it is plausible that longer rest periods may be particularly beneficial for certain populations that perceive a high degree of exertion to sustain higher loads. Additionally, rest intervals ≥2 min may enhance strength gains in resistance-trained individuals [118]. As a general recommendation, the literature suggests a rest interval of 1–2 min between sets is sufficient and that they can be adjusted as needed [91, 117, 118].

With the variables that characterize a single bout concluded, the remaining variables deal with the long-term nature of RT. At the level of a single week, performing multiple bouts per week is advantageous for strength responses, but several meta-analyses concluded that frequency is not a moderating variable for hypertrophy when the training volume was matched between experimental groups [22]. Thus, a variety of volume and frequency combinations can be used to enhance training responses and should be chosen in accordance with what the practitioner and patient can adhere to [22]. Based on the available literature, a minimum frequency of 2 bouts per week is advised to simultaneously enhance hypertrophy and strength responses [22]. In this scenario, an untrained individual might begin with a relatively low volume of 4–6 sets per muscle group per week (i.e., 2–3 sets per bout). As training volume increases, however, distributing the sets across 3 or more weekly bouts may become advantageous.

In line with the above guidance, it’s important to note that the main variables do not need to remain fixed over time, but are often intentionally altered to increase the stimulus in terms of load, volume, and/or frequency. This concept, termed progressive overload, is generally viewed as essential for continuous improvement over weeks to months [22]. Specifically, as training responses occur, absolute loads (i.e., the absolute weight lifted in a given exercise) should be progressively increased to maintain the targeted repetition range at near-maximal effort. We want to emphasize the latter point in this context, where it is conceivable that individuals with FSHD become familiar with RT using low loads for a low number of sets. For instance, an individual with FSHD might choose a frequency of 2 bouts per week, performing a single set per major muscle group using their 25–30RM in weeks 1–3, followed by 2 sets per major muscle group using their 15–20RM in weeks 4–6. After completing this introductory period without adverse events, they could progress to implementing the above recommendations for high-load RT, which have been shown to be the most effective for increasing muscle mass and strength in most other populations. In summary, the described variables should be considered as adjustable tools that practitioners and patients can use to design individualized high-load RT programs, with progression in load, volume, and frequency applied when warranted by the individual’s capabilities, goals, and responses to training [22, 119, 120].

### Primary outcomes of high-load RT

#### Acute responses (hours–days)

Whether a high-load RT program requires further modification can be gauged from the acute responses elicited by a single bout of training. Hence, it is important to understand what the acute responses are that have been observed in healthy populations and how to interpret them appropriately. The first notable responses can begin during the bout and persist for at least a few hours, up to a few days, depending on the program’s variables [121, 122]. Specifically, high-load RT causes rapid neuromuscular fatigue, as indicated by substantial decreases in the rate of force development, as well as the maximal force produced during MVCs [123]. This transient functional decline is also positively correlated with the extent of myofibrillar disruption, as indicated by electron microscopy [123]. These responses are often accompanied by mild muscle swelling for up to 48 h, increased perceived soreness that can last several days, and elevations in serum creatine kinase (CK) levels [124–127]. In addition, acute bouts of high-load RT can transiently increase plasma levels of several cytokines, including IL-6, IL-1ra, MCP-1, and G-CSF, whereas TNFα and IFN-γ, two major mediators of the immune response, remain unchanged [128]. Consistent with the increase in pro-inflammatory cytokines, bouts of RT that induce severe myofibrillar disruption are often followed by mononuclear cell infiltration into muscles [128]. The acute inflammatory responses are regarded as tightly controlled processes that are necessary during remodeling of myofibrillar disruptions [128, 129]. Collectively, these phenomena are usually referred to as exercise-induced muscle damage (EIMD) [128–130].

Importantly, these responses are normal in healthy individuals and must not be mistaken for an intolerance to high-load RT [130]. In fact, it is well established that healthy individuals not only recover from EIMD within a few days, but also appear protected against EIMD in subsequent bouts. This phenomenon is called the repeated bout effect and is based on the observation that the magnitude of the above-described responses is largely attenuated when an individual is subjected to the same bout a second time [129, 131, 132]. Simply put, fatigue and discomfort are expected outcomes in untrained individuals; they are transient and are unlikely to recur to the same extent with repeated bouts.

In line with this protective response to EIMD, high-load RT elicits a powerful anabolic signaling response through mitogen-activated protein kinase (MAPK) pathways and mTORC1, as well as a robust increase in the rate of muscle protein synthesis that persists for several hours to days after the bout [133–140]. Conversely, high-load RT also increases the rate of muscle protein degradation following a bout; however, this response appears to return to baseline within 48 h, whereas protein synthesis may remain elevated beyond this time point [140, 141]. Indeed, the prolonged activation of the anabolic events has been implicated in the ability of skeletal muscle to recover from the myofibrillar disruptions that are caused by a single bout, as well as in the growth and remodeling of skeletal muscle that occurs in response to repeated bouts of high-load RT [139, 142–146].

#### Long-term responses (weeks–months)

To fully appreciate the effects of high-load RT on protein turnover, both muscle protein synthesis and degradation need to be considered. This concept can be illustrated by viewing skeletal muscle as a protein sink, where protein synthesis represents the inflow, and protein degradation represents the outflow. The level of protein in the sink (i.e., muscle mass) is determined by the balance between the two processes: when inflow and outflow are equal, the level of the sink remains stable; when inflow exceeds outflow, the level of the sink increases, and when outflow exceeds inflow, the level of the sink decreases. Applied to high-load RT, it is evident that a single bout acutely increases both inflow (protein synthesis) and outflow (protein degradation); however, the long-term responses suggest that the cumulative increase in protein synthesis exceeds that of protein degradation, resulting in a net accretion of protein/muscle mass [140, 141].

The cumulative effects of high-load RT not only lead to an increase in muscle mass, but also lead to a substantial increase in strength. For instance, 12–24 weeks of high-load RT typically increases an individual’s 1RM by 20–50%, and the increase in strength usually precedes detectable changes in muscle mass [147–150]. While this may seem counterintuitive, the early increases in strength are primarily driven by an increased neural drive, with motor unit recruitment and motoneuron discharge rate increasing within the first 2–4 weeks and contributing significantly to gains in strength [151, 152]. As training continues, morphological changes in skeletal muscle become more prominent, with increases in whole-muscle CSA becoming detectable as early as 3 weeks, and more robust hypertrophy of approximately 5–20% typically observed after 6–24 weeks [147–150, 153]. Of note, these responses exhibit substantial inter-individual variability that appears largely independent of age or sex [147, 148, 154, 155].

Importantly, increases in maximal strength can translate to meaningful improvements in several measures of physical function [22]. For instance, RT has been shown to enhance gait speed, timed up-and-go performance, chair stand performance, and balance in middle-aged and older adults [156–159]. These improvements are particularly relevant for maintaining independence, as they reflect performance in tasks that underpin activities of daily living (ADL) [160]. In summary, high-load RT is a potent therapeutic strategy to increase muscle mass and strength.

In addition to its potential to counter the loss of muscle mass and strength in individuals living with FSHD, evidence from other populations suggests that high-load RT could favorably affect the pathological features of FSHD. For instance, multiple studies have shown that high-load RT reduces fatty infiltration in overweight and/or older adults [161–163]. RT can also attenuate chronic low-grade inflammation, as evidenced by reductions in circulating levels of C-reactive protein, however, effects on pro-inflammatory cytokines, such as IL-6 or TNFα, are less consistent [164–168]. Moreover, RT has been shown to reduce markers of oxidative stress in several metabolic conditions [169]. Finally, several studies have shown that RT alters inflammatory gene expression signatures and reduces interstitial fibrosis in myositis, an autoimmune inflammatory myopathy that, despite being mechanistically distinct, shares many histopathological features with FSHD, including mononuclear cell infiltration, fatty infiltration, fibrosis, and signs of muscle fiber regeneration [170–172].

## High-load RT & FSHD

### Current dogma

Given the body of evidence that suggests that high-load RT has the potential to favorably affect many of the pathological features of FSHD, it would be natural to suspect that high-load RT could be very beneficial for individuals living with the disease. However, when reviewing the literature, we found very little support for the use of high-load RT in individuals with FSHD [173–179]. Instead, there appear to be longstanding concerns—if not dogmatic beliefs—that RT provides no benefit or might even accelerate disease progression [21, 179–181]. As a result, individuals with FSHD have mostly been discouraged from engaging in high-load RT [21]. Specifically, one would find recommendations, such as: i) RT with loads >30% of the 1RM should be avoided, ii) eccentric contractions should be avoided, and iii) persistent pain (> 24 h) after exercise indicates that the individual has overdone it or might be at risk of contraction-induced injury [181]. In other words, there appears to be a major concern that the muscles affected by FSHD may be highly vulnerable to overuse or EIMD [130, 181]. However, as detailed below, our critical review of the literature revealed that the concerns may not be justified.

Before critically appraising the scientific literature underlying these recommendations, we will revisit the long-standing view that muscles affected by FSHD might be highly vulnerable to overuse or EIMD. Our review of the literature revealed that much of this dogma can be traced back to two often-cited studies [180, 182]. Specifically, over 50 years ago, Johnson and Braddom published an article titled “Overwork-weakness in facioscapulohumeral muscular dystrophy”, which has since garnered close to 140 citations [180]. In this study, the authors investigated a family with FSHD and found that most family members were weaker in their dominant arm, which they primarily used during habitual or occupational tasks [180]. This led the authors to conclude that extensive use of muscles, including RT, accelerates disease progression [180]. However, these conclusions were neither substantiated by statistical analyses nor were the methods described in detail [180]. Following up on this, Brouwer et al. studied the relationship of handedness on the strength of the shoulder girdle and the arm muscles in 53 individuals with FSHD and 19 controls [182]. They found that some muscles on the dominant side were weaker than those on the non-dominant side in individuals with FSHD, but not in healthy controls, and this difference was statistically significant in 3 out of 10 tests that they performed [182]. However, when considering the small numerical differences relative to the large standard deviations reported, it is not surprising that later studies were unable to replicate these findings [39, 182–184]. Specifically, two subsequent studies tested MVCs in largely the same set of muscles across a total of 102 individuals with FSHD and found no effect of handedness on side-to-side differences [39, 183]. A case report in 2 pairs of monozygotic twins also reached the same conclusion, and we are not aware of any other studies that have revealed a meaningful prevalence of overwork weakness [184]. Thus, although the studies by Johnson and Braddom and by Brouwer et al. were highly influential, the literature as a whole does not support the notion that muscles affected by FSHD are highly vulnerable to overwork weakness [180, 182].

Having established that the loads lifted during ADLs are unlikely to affect disease progression [39, 183, 184], we searched for evidence specifically supporting the first recommendation: that loads >30% of the 1RM should be avoided. In contrast to this recommendation, we found several RT studies that included individuals with FSHD who repeatedly lifted loads >30% of their 1RM, and all reported that the training was well tolerated [185–189]. This is important because loads of 70–90% 1RM (4RM–12RM) are optimal for maximizing increases in muscle mass and strength, and restricting individuals to lighter loads could needlessly limit the benefits that RT could offer them. However, it should be noted that none of the aforementioned studies directly examined whether muscles affected by FSHD can adequately recover from a bout of high-load RT as described in chapter 3.1. Therefore, future studies should establish whether this is the case using validated markers of EIMD.

The studies cited above suggest that regular RT and ADLs, which inevitably involve submaximal eccentric contractions (e.g., sitting down on a chair), are safe in individuals with FSHD. Given this point, a blanket recommendation to avoid eccentric contractions is implausible. Maximal eccentric contractions, however, warrant separate consideration. To be clear, eccentric contractions are characterized by the lengthening of the muscle–tendon unit under load, and during maximal eccentric contractions, the force-generating capacity of the neuromuscular system is usually far greater (~ 40%) than during maximal concentric contractions (i.e., shortening of the muscle–tendon unit) [190–192]. Moreover, maximal eccentric contractions have a greater capacity to induce muscle damage than concentric contractions, which is why they are commonly used to study EIMD. Thus, for muscles that are highly sensitive to EIMD, the risk may stem not so much from the eccentric mode itself, but from the much higher forces that maximal eccentric contractions permit [129, 130, 193]. We, therefore, wondered whether there is any direct evidence that maximal eccentric contractions should be avoided by individuals with FSHD.

In searching the literature, we found only one study that used maximal eccentric contractions to test whether muscles affected by FSHD are more susceptible to EIMD [194]. In this study, the investigators subjected 2 individuals with FSHD, as well as individuals with other hereditary slowly progressive myopathic diseases, to 2 sets of 8 maximal eccentric contractions of the elbow flexors. Intriguingly, the results revealed that individuals with myopathic diseases showed slightly attenuated signs of EIMD in response to the eccentric bout compared to healthy controls. For example, markers of EIMD, such as serum CK, increased significantly more in the healthy controls 3 days post-exercise. The authors also noted that there were no overt differences in responses between the disorders; however, the study did not include enough subjects to support a firm conclusion about the susceptibility to EIMD in individuals living with FSHD [194]. Thus, submaximal eccentric contractions performed as part of regular RT or ADLs appear to be safe and well tolerated in individuals with FSHD, and the only direct evidence on maximal eccentric contractions, though severely limited by sample size, gives no indication of heightened susceptibility to EIMD. Whether the same conclusion will hold true with the eccentric contractions that occur during high-load RT remains to be determined [185–189, 194].

Lastly, after reading chapter 3.2.1, we hope the reader appreciates that the persistent pain referred to in the third recommendation above summarizes the expected response from any untrained individual who has engaged in RT and is not necessarily an indicator that the individual has overdone it or does not tolerate RT. Needless to say, reports of pain should not be ignored, but we emphasize that exercise and healthcare practitioners should refrain from language that reinforces a sense of fragility, which can contribute to fear and avoidance of exercise [195, 196].

### Studies using (high-load) RT in individuals with FSHD

Given its potential to favorably affect the major pathological features, it is surprising that only a few RT studies have included individuals with FSHD. As mentioned above, all have indicated that RT was well tolerated, and most have reported moderate improvements across a range of strength and functional measures [185–189, 197, 198]. However, most of these studies share critical limitations, such as the lack of FSHD subgroup analyses, very small sample sizes, and/or limited outcome measures, which makes it difficult to generalize the results for individuals with FSHD [185–189, 197, 198].

To date, the most influential study on this topic was published in 2004 by van der Kooi et al., who investigated the effects of RT on the maximal strength of the elbow flexors and dorsiflexors [187]. Specifically, 19 individuals trained these muscle groups on 3 non-consecutive days per week for 52 weeks, while 16 individuals in the control group were instructed to maintain their usual amount of physical activity. The training program started with 2 sets of 5–10 repetitions using a 10RM load, which progressed towards a 5RM over the study duration. In addition, the participants performed a 30-s isometric hold between the 2 sets with the same load used for the sets. Notably, neither the rest interval between the sets nor the hold was described, and no details were offered about the specific exercises that were performed at home with “specially developed training weights”.

Comparing this training program to the recommendations made in Chapter 3.1 reveals several limitations. For instance, the exact exercises that were performed are not known, and it is difficult to determine whether the loads used were truly equivalent to the prescribed repetition maxima since the participants were apparently able to perform a 30-s isometric hold with the same load between sets. As such, it is difficult to assess the likely efficacy of the RT program and whether it would even elicit meaningful training responses in healthy individuals. Indeed, healthy individuals were not included as a positive control group, and thus, any negative findings should be interpreted with considerable caution. That said, the program did produce a 37% increase in the 1RM of the elbow flexors; however, no effect on isometric MVC was observed [187]. Moreover, no significant effects on the 1RM or isometric MVC of the dorsiflexors were found [187].

Over the last 20 years, the van der Kooi study has garnered over 200 citations, and the vast majority of these have emphasized its negative findings [178, 187, 199, 200]. Accordingly, it has been a major contributor to the general consensus that RT offers little to no benefit for individuals with FSHD [173–178]—a consensus that has likely discouraged practitioners from using RT in individuals with FSHD, despite the substantial 37% increase in elbow flexion 1RM and the broadly positive trend across the other studies mentioned above [185, 187–189, 197, 198].

Arguably, whether high-load RT favorably affects the major pathological features of FSHD has not been adequately addressed. With the exception of Bostock et al., none of the available studies utilized high-load RT programs as defined in chapter 3.1 [185–189, 197]. In addition, none of the available studies appropriately considered the asymmetry inherent to FSHD or used gold-standard methods (e.g., whole-body MRI) to investigate its effects on the major pathological features of the disease, such as fibrosis, fatty infiltration, and the loss of muscle mass [185–189, 197]. Thus, until such studies are conducted, clinicians and patients will be left without answers about a potentially beneficial intervention that is readily accessible, low-cost, and likely low-risk.

## Concluding remarks

**Fig. 1:**
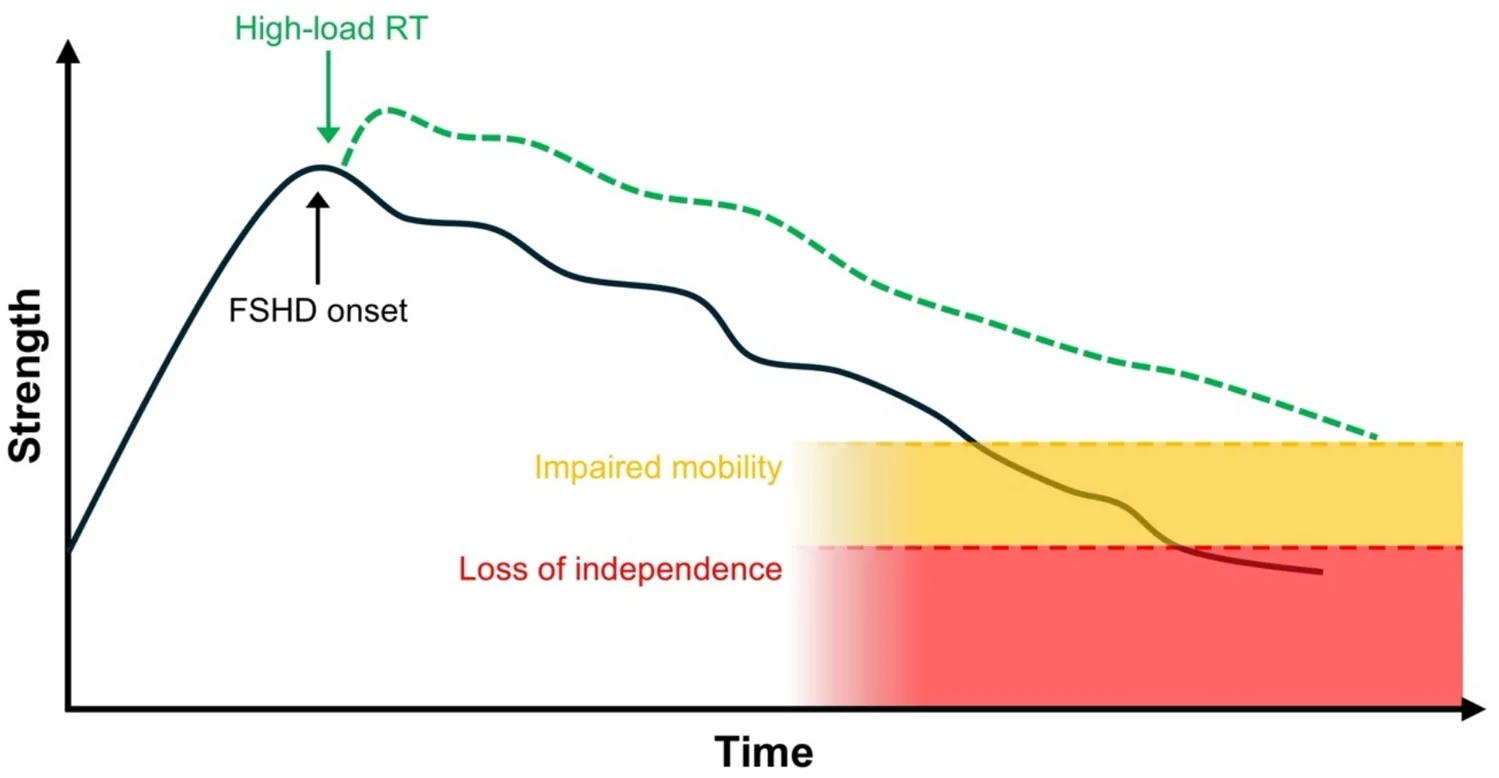
Illustration of how high-load RT could be used to increase strength at the onset of FSHD, thereby delaying or limiting impairments in mobility and the loss of independence in advanced stages of the disease

In this review, we have introduced the basic features of FSHD and presented high-load RT as an exercise modality that has the potential to favorably affect many of the pathological features of the disease. If high-load RT confers the same type of benefits in individuals with FSHD as it does in other populations (e.g., increased muscle mass and strength), then it will be critical to determine if it can also attenuate/delay the impairments in mobility and loss of independence that can occur in advanced stages of the disease (Fig. 1). Accordingly, future studies should establish how disease severity affects the feasibility of high-load RT and/or whether it affects the magnitude of potential benefits.

However, to date, high-load RT has largely been dismissed as ineffective and potentially harmful in FSHD, which has discouraged its use for decades. Our review demonstrates that these convictions lack rigorous support. We, therefore, hope that this review encourages members of the FSHD community to revisit fundamental questions about the safety and efficacy of high-load RT, and to create practical guidelines that are based on rigorous studies.

## Data Availability

Not applicable.
